# Engaging New Parents in the Development of a Peer Nutrition Education Model Using Participatory Action Research

**DOI:** 10.3390/ijerph19010102

**Published:** 2021-12-23

**Authors:** Richard Ball, Kerith Duncanson, Lee Ashton, Andrew Bailey, Tracy L. Burrows, Gail Whiteford, Maria Henström, Rachel Gerathy, Alison Walton, Jennifer Wehlow, Clare E. Collins

**Affiliations:** 1 Mid North Coast Local Health District, Port Macquarie, NSW 2444, Australia; Andrew.Bailey@health.nsw.gov.au (A.B.); gwhiteford@csu.edu.au (G.W.); Rachel.Gerathy@health.nsw.gov.au (R.G.); Alison.Walton@health.nsw.gov.au (A.W.); Jennifer.Wehlow@health.nsw.gov.au (J.W.); 2 Priority Research Centre for Physical Activity and Nutrition, School of Health Sciences, College of Health, Medicine and Wellbeing, University of Newcastle, Callaghan, NSW 2308, Australia; kerith.duncanson@newcastle.edu.au (K.D.); lee.ashton@newcastle.edu.au (L.A.); tracy.burrows@newcastle.edu.au (T.L.B.); maria.henstrom@ki.se (M.H.); clare.collins@newcastle.edu.au (C.E.C.); 3 Health Education and Training Institute (HETI), 1 Reserve Road, St. Leonards, NSW 2065, Australia; 4 Priority Research Centre for Physical Activity and Nutrition, School of Education, College of Human and Social Futures, University of Newcastle, Callaghan, NSW 2308, Australia; 5 School of Community Health, Charles Sturt University, Port Macquarie, NSW 2444, Australia; 6 Department of Biosciences and Nutrition, Karolinska Institutet, 14183 Huddinge, Sweden

**Keywords:** infant, child feeding practices, peer education, social media, participatory action research

## Abstract

This study investigated the implementation model and research methods of a peer education program for new parents focused on infant feeding and nutrition. Two hundred and sixty-nine parents with an infant aged birth to two years old were invited to become co-researchers in a Participatory Action Research (PAR) study over three years. Data included focus group and online participant meeting transcripts, social media data, correspondence between the implementation team and peer educators, and field notes. All data were consolidated regularly and discussed by project participants and the research team. After each PAR cycle, structured content analysis was conducted, informing the next iteration of the implementation model and research methods. Participating parents were highly engaged in child feeding peer-to-peer education, but felt more effective and comfortable being considered as a child-feeding information resource sharer or ‘champion’ rather than a formal peer educator. Similarly, quantitative data collection was only effective when it was integrated seamlessly into the implementation model. PAR methodology suited the diversity and dynamic real-life study setting, facilitating substantial improvements to the peer nutrition intervention model and data collection methods. Our study demonstrated that a genuine collaboration between health professionals and participants to implement research in practice can achieve both intervention outcomes and research aims.

## 1. Introduction

Self-regulation of food intake and dietary preferences develop in the first years of life and can track into adulthood [1]. The diets of Australian children deviate from the recommendation in the national dietary guidelines by the age of two, with an average of 30% of total energy intake from discretionary foods, less than half the recommended number of vegetable servings [2], and total energy intake exceeding requirements by up to 30% [3]. These early-life dietary imbalances have long-term consequences for health and wellbeing.

Parent feeding practices strongly predict the quality and quantity of foods consumed and underpin lifelong eating patterns [4,5]. While parents strongly desire good health for their children, their early intentions about healthy feeding and eating can be thwarted during the preschool years by anxiety around the demands of ‘food parenting’ [6,7] in an increasingly obesogenic environment [8]. Parents who lack child feeding skills tend to resort to feeding strategies that temporarily appease a child, but ultimately result in poor self-regulation of intake, preference for energy-dense, nutrient-poor foods, food anxiety, and avoidance [4,9,10,11,12,13].

Interventions aimed at improving infants’ and toddlers’ eating patterns should guide parents in converting their healthy child feeding intentions into practice [12]. New parents may have limited exposure to evidence-based information to guide the development of feeding practices [14]. They are unlikely to be driven to change feeding behaviour or seek feeding assistance by knowledge of future chronic disease risk [15].

Groups of new parents with similarly aged infants frequently form long-lasting social connections. Along with family, existing friends, and the internet, these peer groups are influential in parents’ child-feeding practices and dietary patterns [16]. Additionally, parents use their peers as a reference point for feeding practices and their children’s dietary intake, rather than health service guidance [8]. Parents’ groups provide the ideal setting for cost-effective, population-level interventions as a group’s social support and norms are resistant to change [17].

Interventions targeting nutrition and feeding practices typically use traditional dichotomies of ‘health professional’ and ‘patient’, yet behaviours are more likely to change if educators share similar demographics and nutritional concerns [18]. Peer-to-peer education is an effective strategy to change health behaviour in some settings [17,19,20,21,22,23] and is likely to influence the naturally occurring knowledge exchange within parent groups. Peer education reduces access issues for rural and remotely located parents, with access further enhanced if peer education interventions capitalise on the online communication pathways used within new parent groups [14]. Parent access to feeding support is particularly important during the COVID-19 pandemic due to the impacts on health service resources, parent isolation, and reported increases in dysregulation of the parent’s own eating behaviours [24].

Our formative work indicated that parents are willing to seek nutrition education for their own purposes and undertake two or more hours of training to become peer nutrition educators [16]. A pilot project in 2014 demonstrated that peer education was acceptable with project participants and the peer groups with whom they shared information. However, the pilot project also indicated a need for additional resourcing to facilitate increased numbers of parents to be trained as peer educators and that the website portal was not a suitable communication platform for parents.

The PICNIC (Parents in Child Feeding Informing Community) program commenced in June 2018 on the Mid North Coast and has trained over 300 participants to date. PICNIC was established to influence the infant and toddler (hereafter referred to as infant) nutrition and feeding social norms within parent networks, taking advantage of newly formed relationships and associating with the naturally occurring information exchange. PICNIC is a genuine example of research in practice, engaging end-users as co-researchers to determine evolving research and an implementation model. Details regarding the PICNIC project protocol have previously been published [25]. To the best of the authors’ knowledge, no current studies exist which have investigated infant and toddler feeding peer education models using informal, existing social structures to share nutrition and child feeding information between parents.

The aim of this study was to address the child feeding needs of rural parents and infants by refining the PICNIC project implementation model and research methods within the overall goal of improving the PICNIC children’s dietary intake and feeding outcomes.

## 2. Materials and Methods

### 2.1. Methodology and Design

This Participatory Action Research (PAR) project was grounded in the research paradigm of pragmatism [26]. The PAR methodology of ‘doing research with us, not on us’ aligns with pragmatism’s philosophical premise that research methods and approaches be practically aligned with the research problem that is being investigated [26]. Recognising the importance of co-production of knowledge through the contributions of participants and researchers, the PICNIC team engaged end-users (parents) as co-researchers [27]. PAR involves cycles of data collection, reflection, planning, and action, identifying required changes, and implementing solutions in real-time. Peer education is ideally suited to PAR as it facilitates participant ownership of modifications to the implementation model [28].

### 2.2. Sampling

The PICNIC project involved purposive sampling of new parents, with progressive recruitment between June 2018 and June 2021. Participants were recruited through health services, early childhood services, social media, and by other parents. Print and electronic posters, advertisements, and videos directed interested parents to the ‘Expression of Interest’ page on the project website [29] where they could access participant information and consent forms. Parents or primary care givers of an infant aged from birth to two years old, who were aged 18 years and older, living within the Mid North Coast Local Health District (MNCLHD), and who were able to understand written and spoken English, were eligible to participate (Table 1). All consenting participants were informed of the project PAR methodology and invited to participate in focus groups and participant meetings, provide feedback on their needs and program experiences, as well as provided opportunities to input into project decision-making. Participants were welcome to contribute as much as they wished, to plan the evaluation of specific program components and to contribute feedback to the research on an ad hoc basis as issues or ideas arose. Methods of communicating ideas included Facebook messenger, phone, email, and meetings. A formal steering committee for the project was not feasible for the new parent participant cohort because parents transitioned out of the early childhood stage within this project’s three-year timeframe.

### 2.3. Data Collection

**Figure 1:**
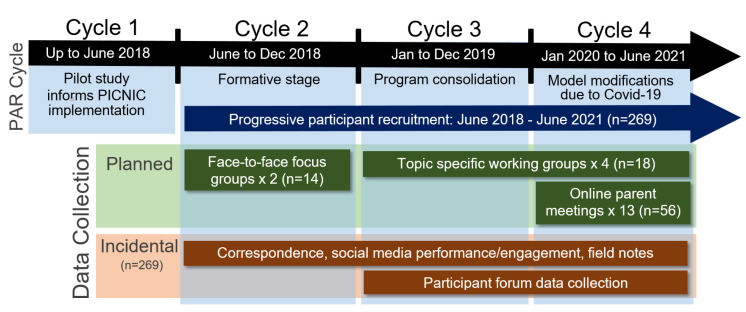
PICNIC PAR cycles, duration, and data collection.

The PICNIC project consisted of four PAR cycles, which are outlined in Figure 1.

Data were collected between, across, and within the four PAR time cycles. Although the cycles are shown in Figure 1 as being sequential and linear, the cyclic nature of PAR meant that cycles overlapped, progressed, and regressed before reaching their endpoint. ‘Planned’ data collection included focus groups, participant meetings, and topic-specific working groups (Figure 1) that were designed to capture participants’ feedback on aspects of their PICNIC experience or program content or structure. ‘Incidental’ data collection was comprised of information volunteered to the research team by participants, observed on social media, or determined from the PICNIC website or Facebook post analytics.

Two face-to-face focus groups (Supplementary Table S1) of approximately 1 h in duration were conducted at health centres in regional New South Wales. Focus groups were conducted by an expert in focus group facilitation and data collection (G.W.), who was not known to participants. Thirteen monthly online parent meetings (Supplementary Table S2) involving between two and eight participants were facilitated by a chief investigator (R.B.) using zoom video technology. These involved bidirectional information exchange between participants (*n* = 56) and the research team. Focus groups and participant discussion groups were audio-recorded and transcribed into Word documents by a professional transcription service. These documents formed part of the dataset that have informed PAR cycle reflections, planning, and action. The focus group data have also been analysed separately for a qualitative research manuscript and are in draft form.

Small groups of between two and four participants were invited to collaborate with the research team to revise the implementation and research components of the project. This included data collection, the social media strategy, and website modifications. All participants were provided with weekly reminders (via a closed social media group) to provide feedback to the research team and make suggestions to inform the content, process, and other program components throughout the intervention period. Incidental data were collated from a range of project-related sources including participant researcher correspondence (messenger, email, phone), observations on the participant forum, and process evaluation of the website and social media platforms, the latter currently being further analysed (using web and social media analytics) and will be reported in a separate paper which is still in draft form.

### 2.4. Data Analysis

Data analysis was conducted both periodically and progressively across the four PAR cycles. Content analysis was undertaken, using ‘planned’ and ‘incidental’ data collection (Figure 1). The planned data analysis involved preliminary analysis of data from the focus groups, online participant meetings, social media data, correspondence, and field notes. Data were manually and independently coded by the chief investigator (R.B.) and associate investigators (G.W., K.D.). The coded data were collated together with incidental data and shared for discussion with participants and the project implementation team to build consensus around the planning and actions for the subsequent PAR cycles and PICNIC project co-design implementation Direct quotes reported in the ‘Findings’ section were representative of participant’s responses in topic areas. The research team met monthly to discuss and reflect on ‘incidental’ data from field notes, social media, participant correspondence, and topics specific working groups. These discussions resulted in the prioritisation and documentation of improvements to the implementation model or research processes that were then raised in participant forums and actioned if feasible within the project resources.

### 2.5. Reflexivity

The chief investigator and research team have extensive health promotion, dietetic, and children’s nutrition work experience. Parents contributed recent lived experience in child feeding and between-parent communications. The de-professionalised nature of PAR facilitated a power-sharing dynamic between the research team and participants, in the co-development of the PICNIC model.

### 2.6. Ethics Approval

Approval for the project was obtained from North Coast New South Wales Human Research Ethics Committee HREC Ref Number: LNR179 (4/12/17) and the University of Newcastle Research Integrity Unit, Ref Number: LNR/17/NCC138(20/10/20). This project has been submitted for registration with the Australian New Zealand Clinical Trials Registry (ANZCTR): Record No. 382430.

## 3. Findings

The PICNIC PAR project commenced in 2018, with the first research cycle used to plan and enact previously identified improvements to the PICNIC implementation model. Since 2018, the project has undergone three major PAR cycles, with cycle 2 defined by the transition from the pilot project to the ongoing PICNIC program, cycle 3 by changes to the model based on participants’ feedback, and cycle 4 defined by the need to move PICNIC completely online in 2020 due to restrictions triggered by COVID-19 (Figure 1). PICNIC recruited 269 participants to the end of PAR cycle 4 (currently 306 participants), with expressions of interest increasing by 78% and recruitment rates by 35% between PAR cycles 3 and 4. PICNIC is currently resourced by 1.8 full-time staff and has streamlined data collection and increased social media reach and engagement; the public PICNIC Facebook page followers are five times the number of PICNIC participants (*n* = 1705). The key PAR stages and changes to PICNIC implementation (participant experience) and modifications to the research model are summarised in Supplementary Tables S3 and S4. The findings are reported in relation to the theme and are an amalgam of all data collected.

### 3.1. Implementation Model Modifications

#### 3.1.1. Peer ‘Sharing’ Enhances ‘End User’ Child Nutrition Information Uptake

The PICNIC implementation model was developed to align with the natural information sharing phenomenon within new parent groups [14]. This facilitated a seamless nutrition information sharing process. However, the co-design team realised in early PAR cycles that the clear delineation between peer educators and education recipients within the PICNIC model was unrealistic. A high proportion of participants recruited as education recipients requested training as peer educators or joined the online forum, therefore receiving the same intervention as peer educators. This indicated a need to reframe the ‘educator’ role towards a ‘champion’ model, in which all parents exposed to the PICNIC intervention became ‘PICNIC parents’. Participants comments about this phenomenon included:

*“I’ve been chatting with the ladies in my mother’s group and a few of them are interested in the PICNIC project”.*

The nature of parents’ social circles was more diverse and dynamic than predicted. In addition to expected contact and information sharing between new parents in their ‘first-time parent’ relationships, there was considerable information sharing within pre-existing friendships and family groups. These recipients were often outside the initial target group age range and geographic area. The online components of PICNIC facilitated information exchange to any geographic location. Examples included:

*“Again, I cannot rate the project highly enough. I’ve even directed my Queensland mum friends to the website”.*

*“My sister even asked me about her five-year-old. He’s quite a fussy eater. I said, go and have a look at the website and just get some hints and tips from there”.*

Parents’ engagement with the peer educator role varied depending on their interpretation of the role responsibilities, their confidence, and the perceived burden of adopting the role. Some parents wanted more nutrition education for their own parenting, while others were keen on ‘educator’ aspects. One participant described her uncertainty about being a peer educator:

*“I felt a bit overwhelmed, like oh okay, now I feel there’s a little expectation that when I’m chatting with mums I need to be, I guess, disseminating information”.*

The research team prioritised the integrity of new parent groups, strengthening peer parent relationships and minimising the research burden in PICNIC project implementation and research. Despite these intentions, the label of peer educators and the formal recruitment of parents (education recipients) was counterproductive. Through targeted discussions in the workshop about how to build evidence-based PICNIC nutrition information into naturally occurring conversations within parent groups, the peer educator label inadvertently added a degree of formality and burden onto participants:

*“You’re going to be a peer educator, it all sounded like a lot more of a commitment than really what it is… without even realising you’re passing information on to people you talk to, just when it comes up in conversation”.*

Reframing the peer educator role as a ‘champion or contact’ in cycle 4 was an effective strategy for resolving parents’ reservations about formally recruiting other parents into PICNIC. Emphasis shifted towards parents sharing experiences and learnings collaboratively. Consuming the same knowledge and implementing feeding practices together strengthened their resolve:

*“So even if you’re not actively trying to spread the word, it still happens to whoever you’re talking to. Really when you’re first starting out those things you’re not talking about much else with other mums, other than what your babies are up to… so you end up sharing a lot of information quite easily”.*

Introductory workshops were originally split into two ages, 6–12 months and 12–24 months, with age-specific content. Introductory workshops were merged for any eligible participants in cycle 3 for logistic reasons. Participants reported that this did not detract from their workshop experience, with parents of older infants happy to be updated and contribute to ‘introducing solids’ discussions and newer parents happy to listen to experiences and insights of other parents.

*“I’m still here. Just taking it all in. It’s good to just listen to all this stuff because you know it’s coming”.*

These findings of peer ‘sharing’ are consistent with Cameron et al. (2010) who highlighted the importance of shared demographics and nutritional concerns as determinants of child feeding behaviour change [18], and Walsh et al.’s (2015) qualitative study findings that peers who had recently introduced complementary foods were the most valued child feeding information source for new parents [30]. The collegial, constructive child feeding network described by PICNIC participants differs from the competitive comparison of child feeding practices between parents of preschool-aged children described by Duncanson et al. (2013) [8]. Future PAR cycles with the PICNIC cohort will determine how parents’ early exposure to the PICNIC child-feeding community influences their child feeding practices and perceptions as children get older.

#### 3.1.2. Impact of COVID-19

Introductory face-to-face workshops were conducted at health facilities from June 2018 until April 2020, when COVID-19 restrictions prevented face-to-face delivery and the workshops pivoted to an online format. This had the unintended benefit of streamlining the workshop part of the intervention. Online workshops can be run more frequently, flexibly, and cost-effectively, with no venue or catering costs and no staff travel time. The online workshops resulted in increased attendance rates, increased partner participation in workshops, and a 47% increase in website usage, now averaging 622 sessions/month. Online workshops have been capped at eight participants to maintain engagement and to encourage interaction and relationship building between participants:

*“I felt welcomed and not judged, I perceived the space as a safe place where I had the freedom to talk about my personal experiences/struggles, creating a sense of belonging, knowing that I was not the only one going through something”.*

Participants in PAR cycle 1 proposed a follow-up workshop or meeting to maintain their focus and obtain more information. These workshops were trialled in cycle 2 but abandoned due to poor attendance. However, once workshops transitioned online, follow-up workshops were re-trialled and now run successfully on a monthly basis. This indicates the demand for additional feeding support and information, and that online delivery meets parents’ needs:

*“Maybe a Three-session, so I could have taken that information away, let it simmer for a little while, and then at least have another one or two opportunities to chat”.*

The fast-tracked emergence of telehealth is cited as one of the few positive consequences of the global COVID-19 pandemic. Benefits of online delivery described by PICNIC participants and the research team are consistent with those reported in a scoping review of telehealth interventions in the first six months of the COVID-19 pandemic [31], from individual child nutrition interventions [32] to a group program for new parents with specific parenting skill needs [33]. The authors of these papers express similar concerns about telehealth including the need for reliable internet access for under-served and rural populations and the evaluation of intervention outcomes to determine their effectiveness.

#### 3.1.3. Digital Platforms for Sharing Evidence-Based Nutrition Information

The project-specific PICNIC website was developed because participants in the pilot project wanted a ‘one-stop-shop’ to access evidence-based, PICNIC-aligned feeding information [14]. Participants have provided feedback on the website in each PAR cycle. Suggestions for improvement have included consistency of the ‘look and feel’ with the PICNIC philosophy. For example, images of participants and their children were preferred to stock photos. In PAR cycles 1 and 2, website navigation/user experience was also identified as needing improvement:

*“I know that the resources are there online, but I guess it’s finding the exact—because there is a lot of information which is good but it’s finding the exact thing that ties in best to be able to share and to show them which is a bit tricky”.*

The PICNIC website originally contained a separate, password-protected page for peer educators, which was replaced by a project-specific closed Facebook group in PAR cycle 2 due to access issues.

*“Don’t create something where we have to go, come to where we are. We are already on Facebook. Most people share everything there”.*

The closed PICNIC Facebook group is used as an information exchange hub and resource repository. Its functions include continued nutrition education, resource provision, and engagement of participants in the PICNIC program review, housing social media posts, and acting as a communication vehicle between parents and the research team. Sharing content via the closed Facebook group facilitates quality assurance because the PICNIC program staff develop and share content for nutrition and child feeding ‘posts’ and can view, edit, and moderate comments using their administrator privileges. The Facebook page, group comments, and analytics provide valuable data about the PICNIC program’s effectiveness and reach. As well as being a trusted participant forum and ’space’, the closed Facebook group effectively alerts participants about new posts. Group members receive alerts for new ‘closed group’ content, whereas public posts compete with all other Facebook content to be seen by the viewer. The functionality of Facebook does not allow closed group content to be shared publicly, which led to PICNIC posts being uploaded simultaneously on the open and closed Facebook pages. Facebook posts are categorised by topic area on the open Facebook page and PICNIC champions invite social contacts, peers, and family to like the public Facebook page. Contacts and friends of participants can be added to the closed group on request.

*“Those little reminders would help me get back on track. No, no, no, we know not to just let him snack throughout the day. We know that there’s six mealtimes a day. Just that—the nice, simple little bits of advice that kept popping up in my feed”.*

To ensure the ongoing relevance of content, it is important for the PICNIC research team members to keep abreast of parents’ changing needs and preferences. An Instagram page was established in response to increasing demand, although Facebook remains more widely used with four to five times more followers. Each social media platform requires slightly different methods and messaging, but also reaches a slightly different audience and increases the overall PICNIC program reach.

Over the last year (cycle 4), 135 people per week have engaged in the Facebook page by liking/commenting/sharing or clicking on posts. Each of the four or five posts shared by the research team each week reached an average of 714 people on Facebook, including both page followers and a wider audience. The social media posts across all platforms have evolved based on performance, engagement, and feedback from participants. Increasing the number of engaged followers helps boost post exposure and performance in addition to post characteristics such as humour, relevance (to child feeding or recent media topics), and visual appeal. For example:

*“I loved the picture that came up recently with the boy clearing the plate. … I shared that one … that got a few laughs”.*

PICNIC participants reported that digital child nutrition interventions should be responsive to participant feedback, meet participants’ needs, and overcome barriers to engagement. These findings align with a recent systematic review on the dietary outcomes and parents’ preferences for digital platform features and functionality [34]. The review reported that parents valued websites with practical tools, engaging and interactive features, a search function, low bandwidth, and minimal setup requirements. In the same review, reported desirable social media platform features were connection and interaction with other users and with health professionals to share information, ideas, achievements, and challenges. The ability to interact with health professionals, post questions, set goals, and receive feedback on progress was also a desired feature. Consistent with PICNIC findings, users had mixed feedback about notifications, reminders, or messaging [34]. Finding a balance between the frequency of posting and notifications without annoying participants will be an ongoing challenge for the research team when sharing on PICNIC social media platforms.

#### 3.1.4. Participant Support Needs

Participant’s engagement, education, and support needs varied widely and seemed to depend more on the parent’s parenting style and personality than nutrition knowledge levels. A high proportion of participants reported being satisfied with the PICNIC ‘intervention dose’ of the two-hour introductory workshop, one-hour follow-up workshop, and closed Facebook group membership with four to five weekly information posts and discussions:

*“I came to the workshop, learned it all, then we chatted in our group and just did it… we all just chilled out”.*

The PICNIC implementation team support roles evolved over the implementation period. PICNIC participants valued the “hands-on” support of the PICNIC program manager and team. This proximity to PICNIC participants allowed the implementation team to attend to participants’ queries and concerns accurately and responsively:

*“Having him [Program manager] behind the wheel of that, he’s so personable and so knowledgeable, and as you [another participant] said, so approachable. I think he was the right person to start that off”.*

The reduced staff time needed to conduct online workshops since mid-2020 has been leveraged to increase time for tailored email or telephone-delivered support to participants who reached out for more education or engagement. Extra PICNIC program information, resources, and health professional referrals were provided, as well as some dietetic-specific services that research team members were qualified to provide. Examples included the provision of reassurance to trust the process of evidence-based feeding practices and managing children’s caution with new foods, their fluctuating appetites, and interest in eating. The PICNIC implementation team also reported that it was valuable and rewarding to invest extra time to build trust and rapport with parents who reached out for more support. Addressing any parent anxiety was critical before feeding practice change was possible:

*“I value these chats because I go through that mum anxiety of, well she’s not eating much, she’s not doing what every other baby is doing. Last time I was feeling that and that really calmed me down to just say, well whatever goes”.*

Parents reported feeling nurtured when the PICNIC team acknowledged their anxiety and uncertainty around child feeding, discussed the challenges of changing feeding behaviours, and provided some guidance on dietetic-specific topics (e.g., food allergies) that are beyond the core PICNIC scope. This responsiveness and understanding enhanced the PICNIC program engagement and uptake.

Investment of the PICNIC team time in building relationships between parents also strengthened the PICNIC program implementation model. The PICNIC group interactions about child feeding built PICNIC parents’ confidence to share within peer parent and social groups. PICNIC team members also acknowledged and valued parents’ expertise and lived experience with child feeding and demonstrated this by having parents answer each other’s questions whenever possible, further instilling confidence to have trust in and to share their knowledge:

*“Then when people did post their personal experiences, you’d have the professional come in and say, yes you’re on the right track, or perhaps look at this. “*

The current status of the PICNIC implementation model at the end of PAR cycle 4 appears to have addressed the majority of the issues identified by parents and staff from the PICNIC pilot and four PAR cycles. This is reflected in comments like:

*“I agree with the whole—about the website, having everything, and then the Facebook feed being that live rolling of throwing in extra points every now and again, and sharing other people’s experiences”.*

These findings of variability in participants’ needs and preferences for the amount and type of support are highly consistent with existing child feeding literature. Collins et al. (2014) have reported associations between parenting style and child feeding practices [5], which is consistent with our finding that addressing feeding-related anxiety needed to occur before participants’ changes to feeding practices were possible. Overall, the implementation model changes have allowed the PICNIC team to reallocate time and resources so that they can meet the needs of the ever-increasing number of participants within existing program resources.

### 3.2. Research Model Transformation

Parallel to the PICNIC model implementation changes over the four PAR cycles, the research methods also evolved between 2018 and 2021. It is envisaged that PICNIC will be completely embedded into the health service practice by the end of 2021 with ongoing data collection conducted for ongoing quality, impact, and outcome assessments. Recruitment and data collection are the two main components of the research model that have changed since 2018.

#### 3.2.1. Participant Recruitment

The original recruitment strategy defined two exclusive participant groups. The research team directly recruited peer educators, who then recruited their peers as education recipients after the introductory workshop. The initial target for participant numbers was 80 peer educators, based on the number of workshops that it was feasible to conduct. It was expected that each peer educator would recruit 2.5 peers, a total of 200 education recipients.

Formal recruitment of education recipients was ceased when there were 35 recipients and 164 peer educators. It was evident that the two-tier recruitment model was not ideal, and that a ‘PICNIC parent’ model which incorporated both groups was more appropriate. Rather than being a flaw in the PICNIC program, the research team considered the overall high engagement with workshops and social media as positive, as it meant the intervention dose received by most ‘PICNIC parents’ was higher than originally envisaged

*“Even those [parents] will still listen to some of the other posts that I shared back in our little mums’ group …so, still like the information, but getting them to join in…”*

The majority of parents were recruited to the PICNIC program when their infant was around six months old. This aligns with when relationships between new parents are established and when parents start introducing solid food to the infants’ diet [35]. Parents reported being comfortable sharing information with peers but uncomfortable recruiting peers as education recipients. Seeking formal consent and encouraging data collection was reported as being detrimental to the natural exchange of parenting and feeding information.

*“… the people that I didn’t ask for a survey, the discussion went really well. The people that had to have that initial muck-around, they were a bit offside at the start”.*

The proportion of referrals to PICNIC by parents (81%) compared to referrals from health professionals and children’s services (19%), greatly increased over the program implementation period. Recruitment promotional activity now focuses on parent engagement, with a social media strategy used for the development and dissemination of recruitment resources. The use of social media as a virtual form of ‘word of mouth’ program promotion has become routine [36] and was effective in PICNIC because participants were positive about their experiences and willing to share:

*“Sometimes hearing another mum recommend a program can be really powerful, for me anyway”.*

The ongoing demand, high recruitment and retention, and ongoing PICNIC program resourcing indicate the PICNIC implementation model has become embedded in health service delivery. As well as meeting parents’ needs, PICNIC is consistent with local health district strategic directions, such as partnering with the community, technology innovation, and obesity prevention [37].

#### 3.2.2. Quantitative Data Collection

Regular contact with participants provided revealing insights into collecting quantitative diet and child feeding data. The research team attempted to use dietary assessment tools that were easy to self-administer, although the participant burden was reported as unacceptable. With a response rate of less than 10%, the 24-h recall and food frequency questionnaire were discontinued after six months and replaced by the Australian Recommended Food Scores for Pre-schoolers [38], a shorter online survey administered 12 months post-intervention. The Feeding Practices questionnaires [39] were relocated to the project website and therefore accessible without the need for a password.

*“I know they’ve just changed the survey style and there’s just one survey now for any age group… that was a good change”.*

Although data collection was not prioritised over PICNIC engagement, the research component of PICNIC was identified as an opportunity to collect longitudinal child feeding data over two to three years and dietary intake data for children who had been ‘PICNIC fed’. This data will be used for impact and outcome evaluation and contribute to pilot data for grant applications and publication of novel findings. With ethics approvals, survey respondents were included in a $100 AUD prize draw for completing all surveys (cycle 3) and provided a $20 AUD grocery voucher for the 12-month feeding survey and ARFS-P (cycle 4). This level of reimbursement is consistent with other studies and is considered adequate to reimburse for time and incentivise participation in this time-poor participant group, without being coercive.

*“Everyone needs groceries. We’re all on mat leave or government pay or something like that. Job Keeper. It all helps”.*

The research team responded to participants’ requests for personalised feedback about feeding practice survey results, so a report template was developed and initiated in PAR cycle 4. While individual report generation takes approximately five to ten minutes per report, this feedback process provided an additional opportunity for intervention, with the feedback report containing links that direct participants to relevant feeding information on the project website. This was recognised as an effective strategy to reinforce feeding information and practices:

*“The questions are a good reminder of the important points right now for us, we need to focus more on family meals”.*

As the PICNIC program transitions from a research project to routine health service practice, child feeding and dietary intake surveys will continue to be administered, but with less intensive follow-up. De-identified data will be used (with consent) to monitor child feeding practices and dietary intake and it is expected that over time this data will provide a valuable, unique dataset that can be used to assess the program and for child health surveillance purposes.

*“I understand it’s a research project … but if that paperwork was completely taken out of the equation, then just the discussion side of it worked really well”.*

Achieving an optimal balance between the collection of data that is meaningful to participants for health service implementation and evaluation and research purposes will be an ongoing challenge for the PICNIC project, as it is in all population-level, translational research [40].

## 4. Recommendations Informing PAR Cycle 5 and an Embedded PICNIC Model

The future direction of the PICNIC program has been informed by the participatory action research in the pilot project and four PAR cycles to date, but some areas of need have not yet been fully addressed. Earlier awareness of PICNIC in the prenatal or very early postnatal stage was recommended, with participants suggesting ways that PICNIC information can be provided to antenatal and maternity staff to share:

*“PICNIC Flyers would be a great inclusion. It would be great to have this information before the baby is even born”.*

To achieve this outcome, the referral pathway will need to be embedded in strategic and clinical child, family, and maternity services plans [40]. The dietetic expertise within the PICNIC team provides an important link in the health service continuum of care because child feeding issues can be triaged effectively for management within PICNIC or serve as a waitlist intervention and referral pathway to clinical dietetic services. The PICNIC implementation and research teams are aware of the importance of reporting on outcomes that are aligned with local and state health priorities for PICNIC program continuity within the local health district and to meet demands for program scaling and adaptation.

Despite participants’ high engagement with the PICNIC program, survey completion continues to be the most challenging aspect of the program, for both participants and the research team.

*“Unfortunately, I didn’t gain much interest at all from my mothers’ group as they have been approached by the other [PICNIC] mums”.*

Administration of the validated child feeding and diet quality scoring tools has been substantially streamlined, with the collection and preliminary analysis of child feeding practices and dietary intake data being undertaken. Quantitative data will be further analysed to test for associations between these variables and PICNIC engagement and used in an economic evaluation being undertaken by the local health district. These outcomes will be published once enough data is collected to be clinically relevant and robust.

In the future, links to the surveys will be added to the PICNIC website home page for ongoing data collection and the automated survey feedback mechanisms will be further enhanced so survey participants get immediate feedback on child feeding and dietary intake. This data will be used to analyse child feeding practices and dietary intake of ‘PICNIC children’, producing population-level dietary information that can be compared with other data collected at a health district level, such as routine height and weight population-level data.

The accessibility of the PICNIC website and social media content increases the accessibility and transparency of this research. This is a strength of PICNIC, in that the content is readily accessible for interested parties to view and critique, but a challenge in terms of maintaining PICNIC program fidelity and intellectual property. There have been expressions of interest from five other local health districts, non-government organisations, international research groups, and businesses to implement or contribute to PICNIC, all of whom have slightly different needs and visions for the program. Adaptations to the PICNIC model that have been discussed include commercialisation of the ‘PICNIC model’, an adaptation of workshops as an online course, and a childcare-specific version of PICNIC to train early childhood educators in the PICNIC approach:

*“It would be a great way to not just reach parents, but educators in childcare settings are exposed to so many parents there, so if they could have the training too”.*

The potential likelihood of scaling PICNIC has resulted in standardisation and documentation of PICNIC processes within the local health district. The outputs from this process have been a PICNIC manual and guidelines for workshop implementation, PICNIC parent engagement, and social media content and posting procedures. For scaling up to other local health districts, development of international versions of PICNIC, commercialisation, or adaptation for different settings (e.g., childcare) or modalities (e.g., online), the PICNIC implementation team is exploring license agreements, intellectual property requirements, and memoranda of understanding. The PICNIC implementation and research teams are motivated to explore such options by the opportunity to broaden the reach of PICNIC, especially when current ‘PICNIC parents’ provide feedback like:

*“Also just wanted to commend the project/research design. My partner and I both have academic backgrounds and it’s great to see a research project that is not just ‘taking’ from participants but is really giving in the form of education and aiming to change behaviour in a positive way, while being transparent about its methods. ”*

## 5. Conclusions

New parents were highly engaged in seeking information about child feeding and disseminating key learnings via their peer networks. Parents were more comfortable considering themselves as a ‘PICNIC parent’ or ‘feeding champion/contact’ rather than a peer educator, reporting that this aligned with the organic nature of information sharing within social groups. The PAR methodology employed in the PICNIC project contributed to the progressive and responsive adaptation of the intervention model and research methods. The pragmatically framed PAR suited the diverse and dynamic real-world setting, catering to diverse demographics, geographic locations, and parent support needs. Participants strongly supported the use of communication platforms and methods that are widely accessed by the target demographic. Our findings emphasise the importance of working closely with program participants to design effective and acceptable feeding interventions for new parents. They also highlight that careful attention to study design is required to ensure unobtrusive research methods, which do not undermine the intervention. The PICNIC project shows promise as a peer support intervention to influence parent feeding practices. Future analysis of child feeding and dietary intake data will determine whether PICNIC influences the nutritional and health status of children in rural Australia.

## Figures and Tables

**Table 1:** Parent/Care giver demographic characteristics of participants in the PICNIC project (total *n* = 269).

**Gender**		**Age-Range**		**Indigenous Status**		**Attend Parent Group**	
Male	5 (2%)	18–24 years	19 (9%)	Indigenous Australian Non-Indigenous Australian	11 (5%)	No	55 (26%)
Female	261 (98%)	25–34 years	138 (68%)		196 (95%)	Yes	155 (74%)
		35–44 years	47 (23%)				
**Highest Education Level**		**Employment Status**		**No. of Children**		**Youngest Child**	
Year 10 or equivalent	9 (4%)	Not employed	38 (18%)	1 Child	159 (76%)	0–6 months	104 (49%)
Year 12 or equivalent	46 (22%)	Maternity leave	100 (48%)	2 Children	35 (16%)	6–12 months	70 (33%)
Trade/Vocational	25 (12%)	Part-time employed	45 (22%)	3–5 Children	16 (8%)	12–24 months	37 (18%)
University degree	122 (59%)	Full-time employed	24 (12%)				
Other	6 (3%)						

## Data Availability

The data presented in this study are available on request from the corresponding author.

## Supplementary Materials

The following are available online at https://www.mdpi.com/article/10.3390/ijerph19010102/s1, Supplementary Table S1, PAR Cycle 2: Focus Group Questions. Supplementary Table S2, Online Parent Discussion Outline. Supplementary Table S3, PICNIC implementation model modifications. Supplementary Table S4, PICNIC research methods modifications.
