# Supplementary material for: Engaging New Parents in the Development of a Peer Nutrition Education Model Using Participatory Action Research
Source: Int J Environ Res Public Health. 2021 Dec 23;19(1):102. doi: 10.3390/ijerph19010102 (PMC8750105; doi:10.3390/ijerph19010102)
Supplement: Supplementary file 1 [file ijerph-19-00102-s001.zip › ijerph-1455536-supplementary/Supplementary Table S1.pdf]

Supplementary Table S1. PAR Cycle 2: FOCUS GROUP QUESTIONS.

1. What motivated you to become involved in PICNIC?
2. In what ways was it what you thought it would be or in what ways was it different from how you thought it would be?
3. What have YOU got out if it?
4. What do you think OTHERS get out of it?
5. What are the key aspects of the PICNIC approach that seemed to work? e.g. timing, sharing?
6. Can you tell me about your experiences of feeding your children after being involved in PICNIC has changed, or your expectations of what feeding will be like?
7. What was it like for you to be an educator in PICNIC?
8. How did it make you feel about yourself and other parents?
9. What knowledge and skills did you develop?
10. If someone were to ask you why they should get involved in PICNIC, what would you tell them?
11. Any other thoughts or comments?
