# Supplementary material for: Engaging New Parents in the Development of a Peer Nutrition Education Model Using Participatory Action Research
Source: Int J Environ Res Public Health. 2021 Dec 23;19(1):102. doi: 10.3390/ijerph19010102 (PMC8750105; doi:10.3390/ijerph19010102)
Supplement: Supplementary file 1 [file ijerph-19-00102-s001.zip › ijerph-1455536-supplementary/Supplementary Table S2.pdf]

Supplementary Table S2. Online Parent Discussion Outline.

**Introduction**	• Welcome to country<br>• Thank you and describe the group i.e. a mix of ages and therefore some younger ones will get some insight into what to look forward to.<br>• Describe the session, driven by participants<br>• Go around group: introduce yourself, bub and have a chat re/ your feeding journey, where you up to and anything you'd like to discuss here in the group.
**Q and A**	• Discussion period
**Feeding Prompt Questions**	• How do you think you feeding is going?<br>• Anything you need to clarify from the workshop?<br>• Which were the most helpful topics the peer educator shared with you?<br>• Did you join at the right time for the age of your child?<br>• How is your feeding was compared to how you though it might be?<br>• Describe an occasion where you used information for the benefit of your child.
**Peer Education**	• Discuss the nature of peer education, the project and ask how it worked for you.<br>• Anyone shared anything yet with friends who haven't attended<br>• Talk about the group dynamics, more or led interested parents.<br>• How do you think the information was received by other friends in your group?<br>• What is your confidence like to share info?<br>• Family/grandparents experiences?<br>• Conflicting information, hasc anyone had messages challenged?<br>• What would've helped better support you sharing with other parents.
**Support resources**	• Website usage<br>• Social media usage<br>• Closed group/ open page<br>• How well (or not) was the information provided pitched for you?
