# Supplementary material for: Engaging New Parents in the Development of a Peer Nutrition Education Model Using Participatory Action Research
Source: Int J Environ Res Public Health. 2021 Dec 23;19(1):102. doi: 10.3390/ijerph19010102 (PMC8750105; doi:10.3390/ijerph19010102)
Supplement: Supplementary file 1 [file ijerph-19-00102-s001.zip › ijerph-1455536-supplementary/Supplementary Table S3.pdf]

Supplementary Table S3. PICNIC implementation model modifications.

Participatory Action Research cycles in the PICNIC project, including four stages of model implementation refinements

Participatory Action Cycle	Cycle 1: Pre-Jun 18 (Changes to model after pilot project)	Cycle 2: Jun – Dec 18 (Formative stage)	Cycle 3: Jan – Dec 19 (Program Consolidation)	Cycle 4: Jan 20 – Jun 21 (Model changes due to COVID-19)
**Distinguishing Peer Educators and Education Recipients**		Peer Educators and Education Recipients considered mutually exclusive	Education recipients beyond Mid North Coast Local Health District geographic area and older than 0 to 2 age group	Peer educators and education recipients combine to become PICNIC parents<br>Education recipients consenting to be peer educators.<br>Education recipients joining Closed Facebook Group
**Project workshops**		Participants allocated to one of two Introductory workshops based on infant age (0-12 or 12-24 months)	Introductory workshops (0-12 or 12-24 months) merge into one workshop: infant age 0-24 months<br>Additional follow-up face to face workshop trialled following requests, discontinued due to low uptake	Two-hour Introductory workshop moved online (COVID -19 restrictions)<br>One-hour follow-up workshops trialled online and sustained
**Website**	PICNIC project website created. Project specific nutrition/ feeding information for infant/ children 6 to 36 months	Dietetics student projects: website changes in response to feedback and identified needs	Dietetics student projects: website changes in response to feedback and identified needs	Website upgraded with addition of news feeds and search-tag system for navigation. Compliant with NSW Health Website Guidelines [34]
**Social Media Strategy and online participant forum**	Online participant forum developed and housed on Password protected portal in PICNIC website alongside Social Media posts to share	Online participant forum moved from website portal to Facebook Closed group (PICNIC Peer educators). Participants invited to join at Introductory workshop<br>Post/messages shared to Facebook closed group and Facebook public page concurrently<br>Posts organised into folders on the public Facebook page<br>Stock images replaced by images provided by participants	Participants request access to and invite parents within their social network to the online participant forum (Facebook closed group)	PICNIC Peer educators (closed Facebook group) renamed PICNIC parents<br>Instagram page created Post/messages shared to Facebook closed group, Facebook public page and Instagram pages concurrently<br>Consideration of future additional PICNIC Social media platforms (e.g., Tik-Tok)
**Participants' child feeding support needs**		Support provided outside core PICNIC content (i.e., allergies) requested by participants, (within PICNIC platform or individually by phone or email)	Continued engagement and support requested by participants outside 12-month intervention period	Support for issues outside core PICNIC content built into online participant meetings<br>Extension of age requested and PICNIC 3-6 model in Development
