# Supplementary material for: Engaging New Parents in the Development of a Peer Nutrition Education Model Using Participatory Action Research
Source: Int J Environ Res Public Health. 2021 Dec 23;19(1):102. doi: 10.3390/ijerph19010102 (PMC8750105; doi:10.3390/ijerph19010102)
Supplement: Supplementary file 1 [file ijerph-19-00102-s001.zip › ijerph-1455536-supplementary/Supplementary Table S4.pdf]

Supplementary Table S4. PICNIC research methods modifications.

Participatory Action Research cycles in the PICNIC project, including four stages of modifications to research methods.

Participatory Action Cycle	Cycle 1: Pre-Jun 18 (Changes to model after pilot project)	Cycle 2: Jun 18 – Dec 18 (Formative stage)	Cycle 3: Jan – Dec 19 (Program Consolidation)	Cycle 4: Jan 20 – Jun 21 (Model changes due to COVID-19)
**Participant Recruitment**		Peer Educator recruitment: Antenatal/Child and Family Health/ Childcare/Facebook and by snowball<br>Participants recruit education recipients after Introductory Workshop<br>Recruitment aim: 100 Peer Educators and 200 Education Recipients in one year<br>Recruitment period: One year	Prioritisation of Facebook and parent to parent recruitment over Health Service referrals<br>Recruitment aim: 150 Peer Educators and 150 Education Recipients (more direct involvement)<br>Recruitment period: Ongoing	Recruitment period: Ongoing for 3 years with transition into practice (300 participants)<br>Progressive recruitment with project embedded in usual care with the health district<br>Recipient recruitment embedded in implementation
**Quantitative data collection**	Feeding practice survey (FPSQ) located on password protected portal within PICNIC Project website	Feeding practices (FPSQ) 24hr recall (ASA-24) and food frequency (AES) collected from Peer educators and Education Recipients at the of intervention start, six and 12 months	ASA-24 and AES ceased and replaced ARFS-P<br>Peer educators and Education recipients complete feeding practices survey (FPSQ) at intervention start, six and 12 months and diet quality survey (ARFS-P) at 24 months (12 months post intervention)<br>Up to two reminder text messages (with survey link) sent to participants	Data collection (FPSQ) at 0 and 6 month time points removed for education recipients due to recruitment issues<br>Data is collected from Peer educators and education recipients morphing as one participant group
**Incentives**		Participant pack includes cooler bag, plate, spoon, bib, branded infant shirt	Participant pack includes infant shirt only<br>$100 grocery voucher (monthly raffle draw) entry for completion of surveys at 0, 6 and 12-month intervention timepoints	$20 grocery voucher provided to participants for completion of 12 survey at 12 months (FPSQ) and ARFS-P surveys<br>Personalised feeding report provided for each feeding practice survey completed
**Project growth, scaling, external interest and partners**		Need for funding identified and economic evaluation to support grant applications identified	Partnerships with child feeding researchers from Sweden, Collaboration initiated with interested additional Local Health Districts (LHDs)<br>Need identified for related and reinforcing content for people and organisation's looking after children	Economic evaluation data collection commenced for Cost Consequence Analysis and Health Impact Assessment<br>Collaborations with other LHDs established<br>Scale up implementation model being developed

Note: FPSQ = Feeding Practices and Structure Questionnaire, Milk and Solids Version [35], **ASA-24 =** Automated Self-Administered 24-Hour (ASA24®) Dietary Assessment Tool [36] **AES** =Australian Eating Survey: Food Frequency Questionnaire, University of Newcastle [37], **ARFS-P**= Australian Recommended Food Scores for Pre-schoolers [38].
